# Supplementary material for: Molecular mechanism and structural basis of small-molecule modulation of the gating of acid-sensing ion channel 1
Source: Commun Biol. 2021 Feb 9;4:174. doi: 10.1038/s42003-021-01678-1 (PMC7873226; doi:10.1038/s42003-021-01678-1)
Supplement: Supplementary file 2 — Supplementary Information [file 42003_2021_1678_MOESM2_ESM.pdf]

Molecular mechanism and structural basis of small-molecule modulation of the gating of acid-sensing ion channel 1

Yi Liu[1*], Jichun Ma[2], Renee L. DesJarlais[2], Rebecca Hagan[1], Jason Rech[3], David Lin[3], Changlu Liu[1], Robyn Miller[2], Jeffrey Schoellerman[1], Jinquan Luo[4], Michael Letavic[3], Bruce Grasberger[2] & Michael Maher[1]

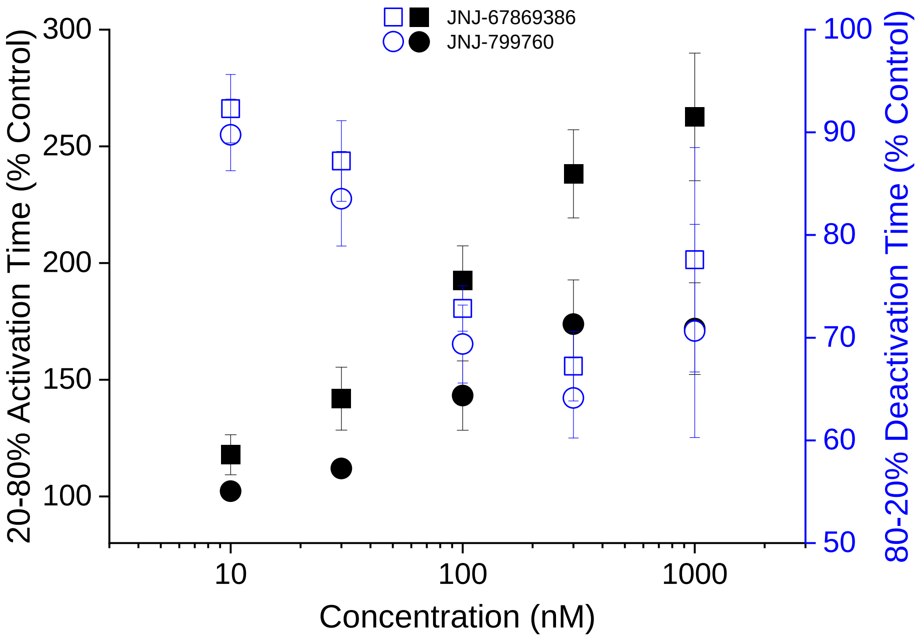

Supplementary Figure 1. Rate of channel activation and deactivation in the absence and presence of JNJ-67869386 or JNJ-799760. The 20-80% activation times (solid symbols) and 80-20% deactivation times (open symbols) are normalized to control values (in the absence of compound) for each cell before averaging. Data are statistically different from control for the following groups: 100 nM ($p<0.01$), 300 nM and 1000 nM ($p<0.001$) for JNJ-67869386 activation (n=4); 100 nM and 300 nM ($p<0.01$) and 1000 nM ($p<0.05$) for JNJ-67869386 deactivation (n=4); 300 nM and 1000 nM ($p<0.01$) for JNJ-799760 activation (n=4); 30 nM

(p<0.05), 100 nM (p<0.01), 300 nM and 1000 nM (p<0.001) for JNJ-799760 deactivation. All statistical tests are One-way ANOVA. Holding pH=8.2. Test pH=6.0.

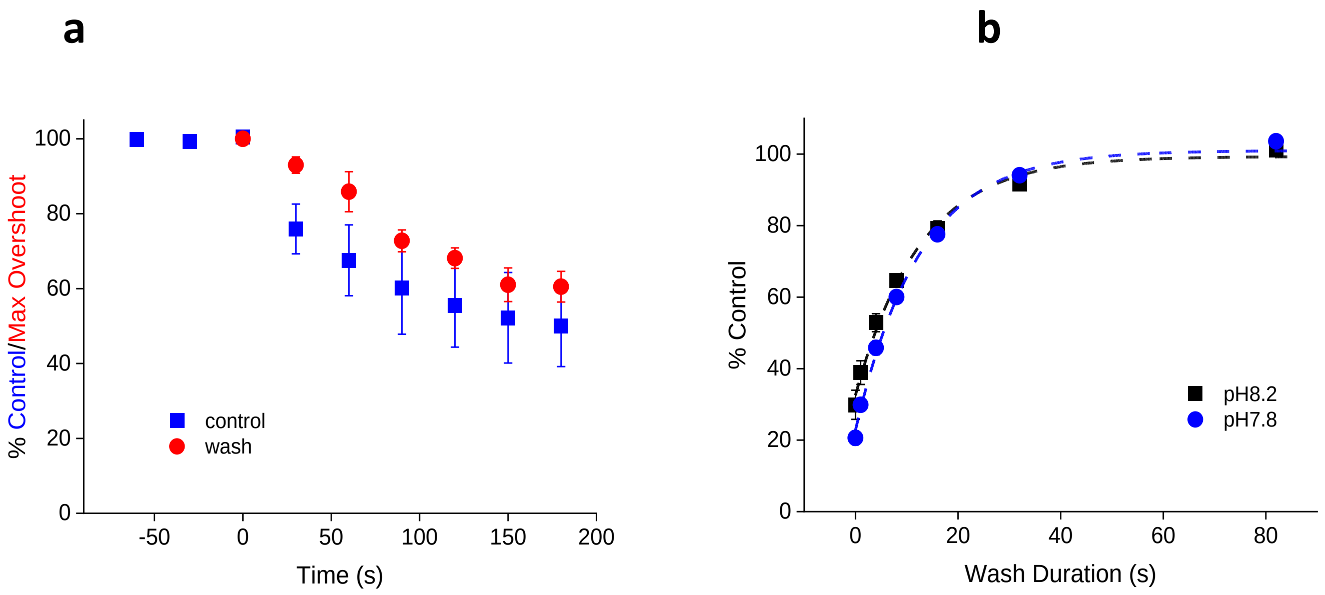

Supplementary Figure 2. Development of and recovery from closed-state desensitization. (a) Development of pH 7.4-induced desensitization. For control cells (squares), the holding pH is switched at t=0 sec from 8.2 to 7.4. The current amplitude is normalized to pH8.2 (n=4). For JNJ-67869386 wash cells (circles; holding pH=7.4 throughout experiment), the maximal overshoot current amplitude is set to occur at t=0 sec and used to normalize subsequent currents (n=4). Test pH=6.8. (b) Kinetics of wash at pH 8.2 (squares) or pH 7.8 (circles) from inhibition by 100 nM JNJ-67869386. pH 6.0-evoked currents are normalized to pre-compound control. Data are fitted to a single exponential function with time constants of 12.7±1.3 sec (pH 8.2; n=5) and 12.6±0.6 sec (pH 7.8; n=3), respectively, and are statistically indistinguishable between the two pHs ($p>0.3$; Two-way ANOVA).

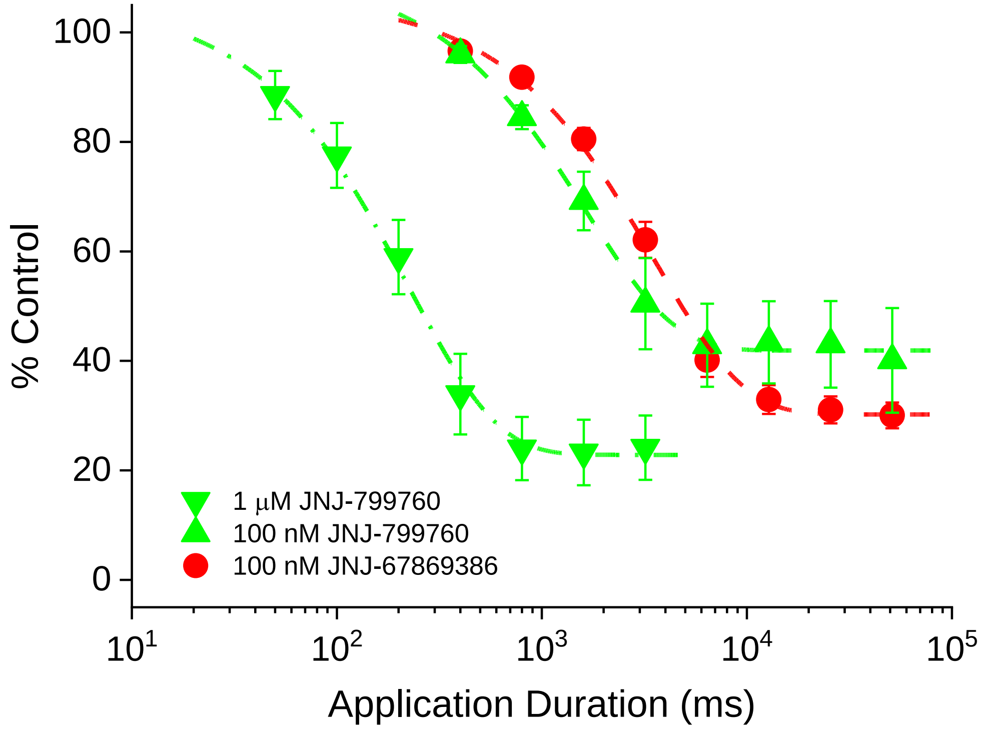

Supplementary Figure 3. Kinetics of current inhibition by JNJ-67869386 and JNJ-799760. A series of brief pH 6.0 test pulses are interspersed throughout the compound application period. Holding pH=8.2. Data are normalized to the pre-compound pH 6.0 peak and fitted to a single exponential function with time constants of 3.6±0.2 sec (100 nM JNJ-67869386; circles; n=9), 1.6±0.1 sec (100 nM JNJ-799760; triangles; n=4; p<0.01 vs JNJ-67869386; Two-way ANOVA), and 224.8±18.9 ms (1 µM JNJ-799760; inverted triangles; n=4; p<0.001 vs 100 nM JNJ-799760; Two-way ANOVA), respectively.

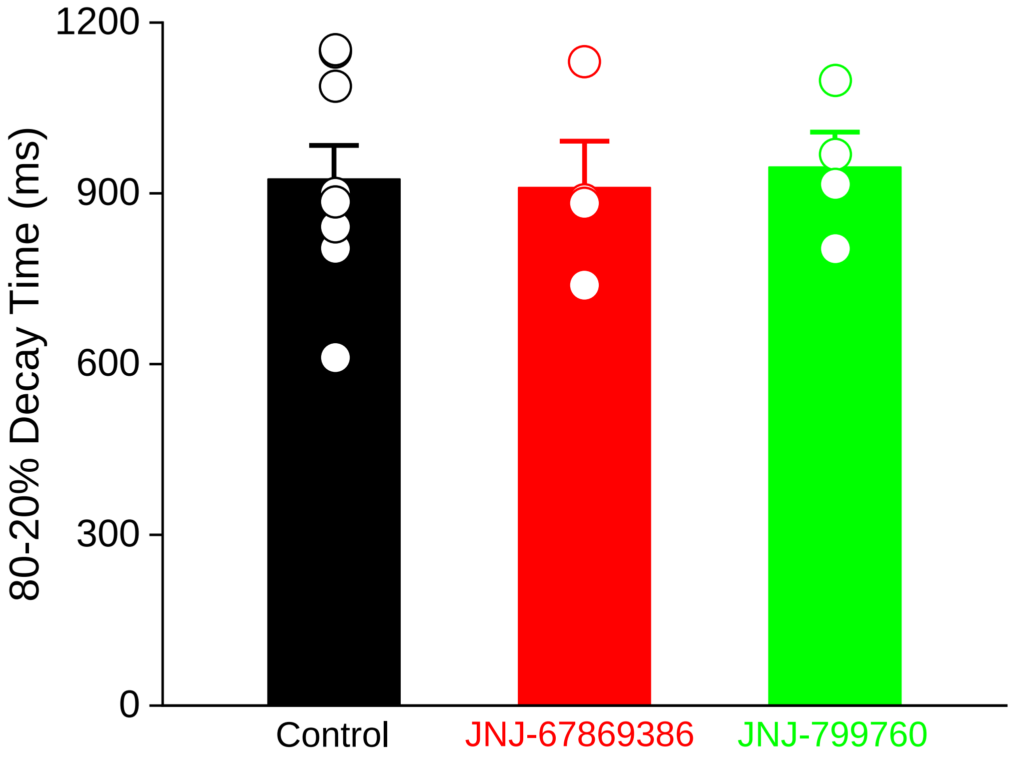

Supplementary Figure 4. Decay time (80-20%) of pH 5.0-induced desensitization. Summary for all experiments in Fig. 5d in which compound is present only during the pH 5.0 application period (n=9, 4 and 4 for control, 100 nM JNJ-67869386 and 100 nM JNJ-799760, respectively; p>0.8 vs control for both compounds; One-way ANOVA). Data for individual cells are shown in open circles.

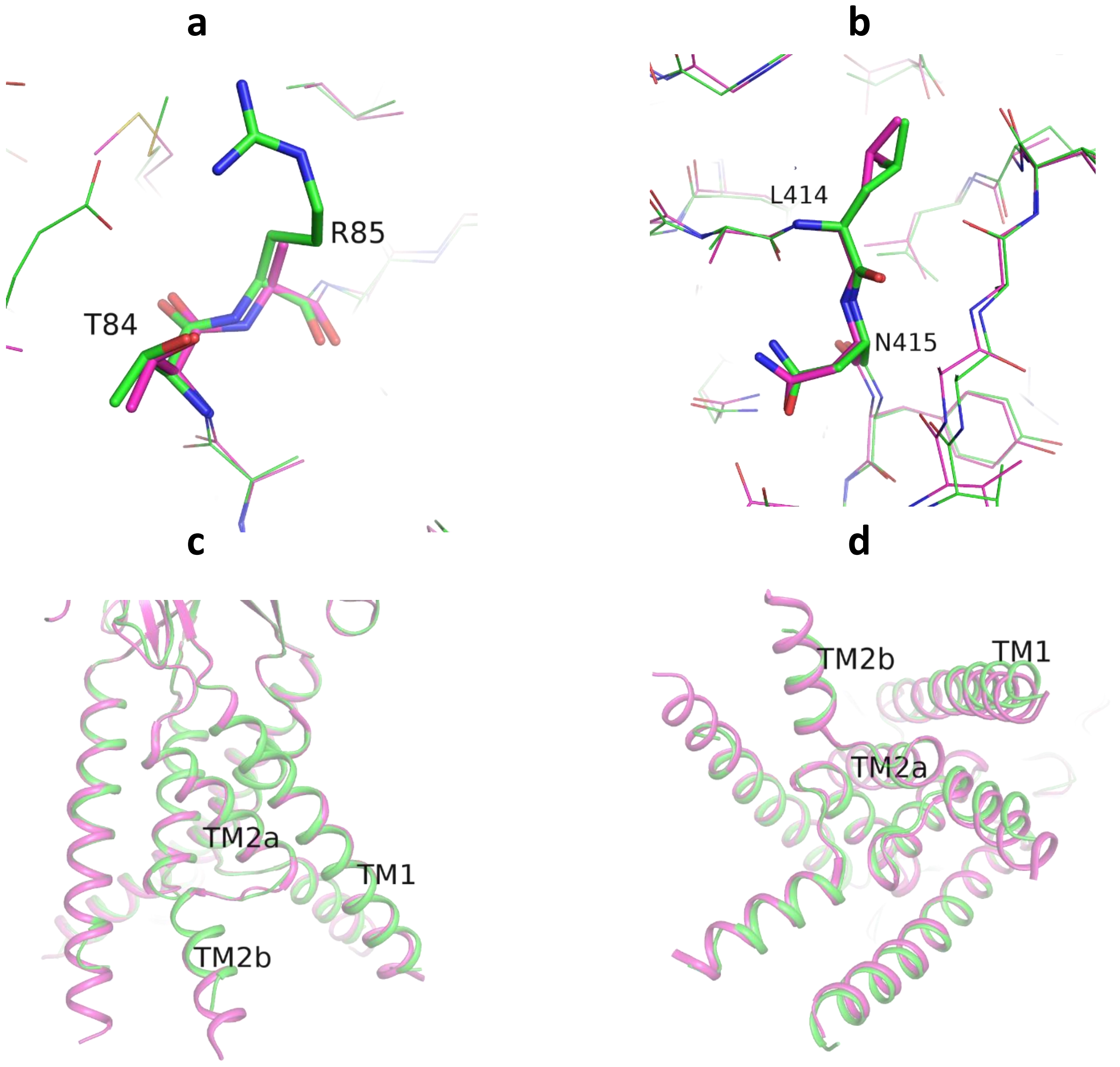

Supplementary Figure 5. Overlay of key channel conformations of ΔASIC1/JNJ-799760 (green) and 5WKU (pink), a closed-state structure. (a) Orientation of the T84-R85 peptide bond. (b) Orientation of the L414-N415 side chains. (c) GAS belt extension and TM2b domain swap. (d) Dimensions of the channel gate viewed from the intracellular side.

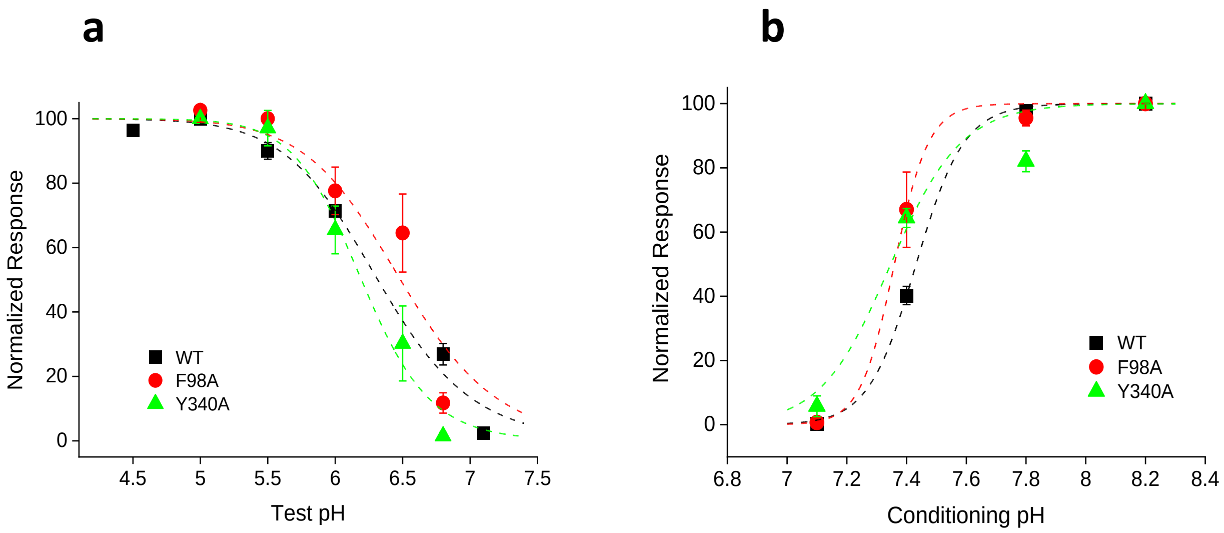

Supplementary Figure 6. pH dependence of activation and steady-state desensitization of rASIC1a WT and mutant channels. (a) pH dependence of activation. Current amplitude is normalized to that of pH 5.5 (F98A) or pH 5.0 (WT and Y340A) for each cell before averaging. Holding pH=8.2. pH_{50} values from the best fits (dashed lines) are: 6.31±0.07 (WT; n=11), 6.48±0.12 (F98A; n=4), and 6.19±0.05 (Y340A; n=4), respectively. Data for the mutant channels are statistically indistinguishable from WT (p>0.05). (b) pH dependence of steady-state desensitization. Current (evoked by brief pulses of pH 6.0 or pH 5.5) amplitude is normalized to that at holding pH 8.2 for each cell before averaging. pH_{50} values from the best fits (dashed lines) are 7.43±0.01 (WT; n=14), 7.36±0.03 (F98A; n=4; p<0.05), and 7.35±0.05 (Y340A; n=4; p>0.05), respectively. All statistical tests are One-way ANOVA.

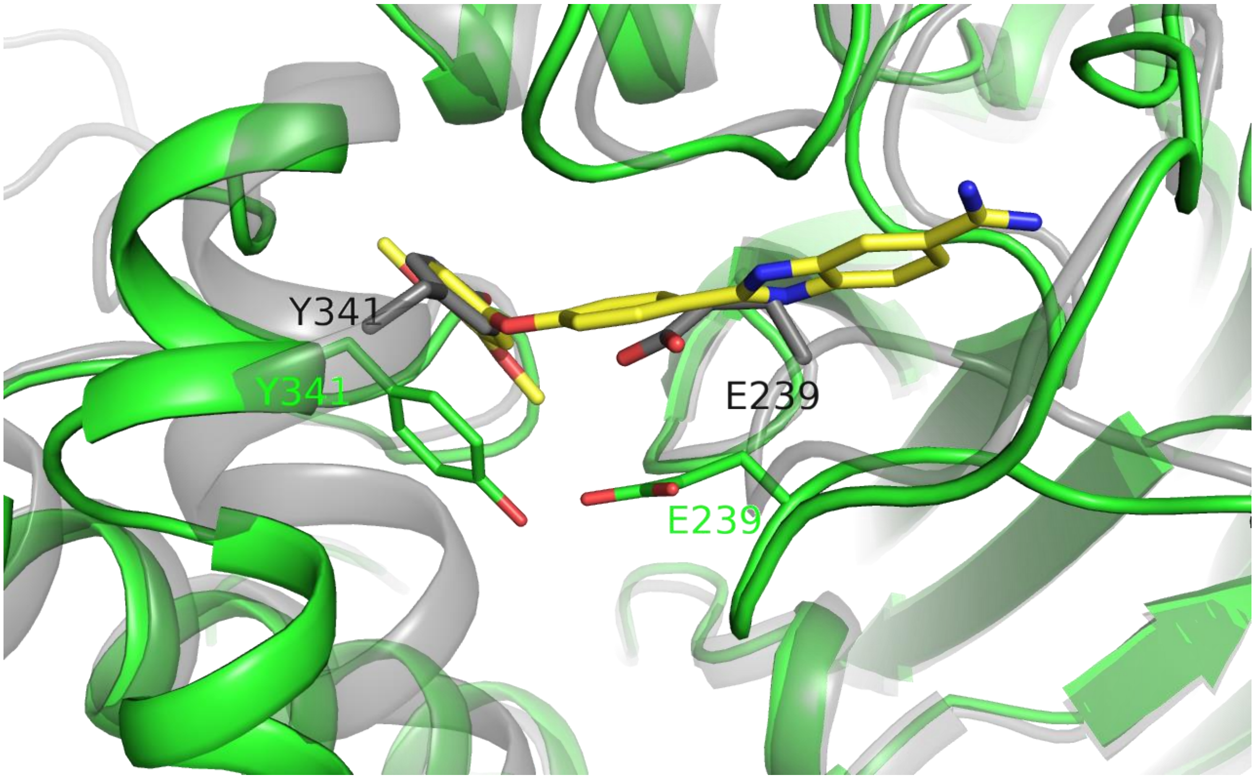

Supplementary Figure 7. Overlay of ΔASIC1/JNJ-799760 with 2QTS at the JNJ-799760 binding pocket. Note that E239 and Y341 in 2QTS (gray) are in direct steric clash with JNJ-799760 in ΔASIC1/JNJ-799760 (green).

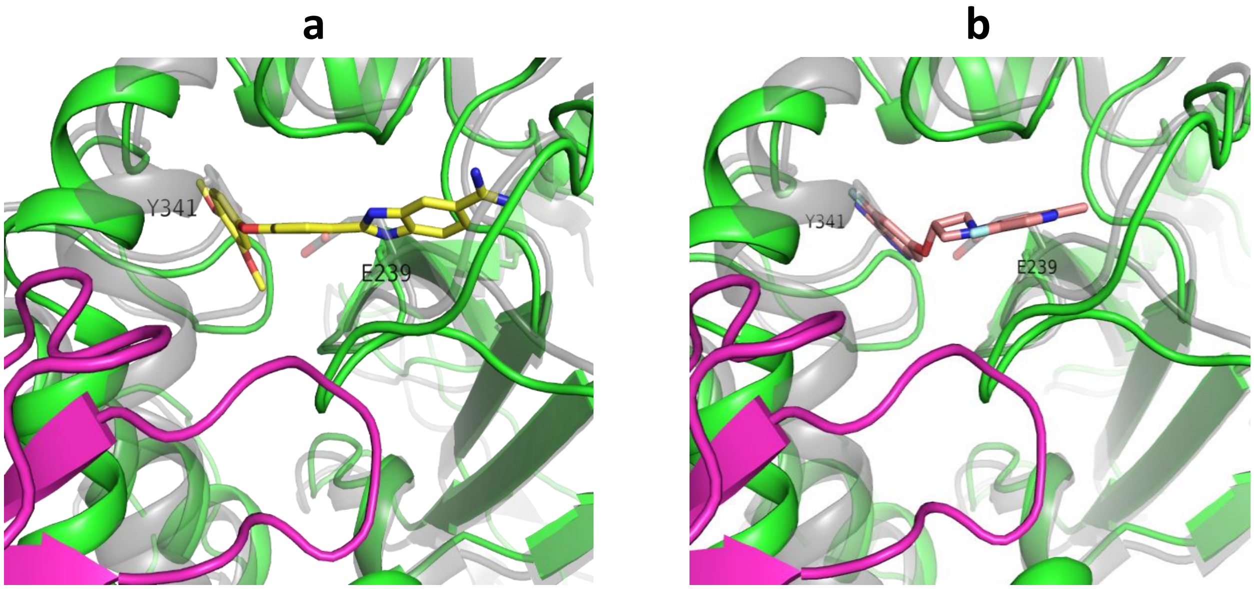

Supplementary Figure 8. Comparisons of JNJ-799760-, JNJ-67869386- and PcTx1-bound ASIC1 structures near the acidic pocket. (a) Overlay of ΔASIC1/JNJ-799760 (green/yellow) with ΔASIC1/PcTx1 (3S3X; gray/magenta). Note that E239 and Y341 in 3S3X are in direct steric clash with JNJ-799760 in ΔASIC1/JNJ-799760. A portion of the PcTx1 molecule is shown binding to α5 and protruding into the acidic pocket. (b) Overlay of the ΔASIC1/JNJ-67869386 docking structure (green/salmon) with 3S3X. Note that E239 and Y341 in 3S3X are in direct steric clash with JNJ-67869386 in the docking structure.

Channel State	Key Conformations					PDB ID/Reference
	Gate Size*	Acidic Pocket	T84-R85 Bond Flip	L414-N415 Side Chain Swap	GAS Extension/TM2b Swap	
Open	Wide	Collapsed	Yes	Variable	Variable	4NTW[1], 4NTX[1], 4NTY[1], 3S3X[2], 4FZ0[3], 4FZ1[3]
Desensitized	Narrow	Collapsed	No	Yes	Yes	3IJ4[4], 4NYK[4], 6CMC[5], 6VTK[6]
Desensitized-like	Wide	Collapsed	No	Yes	No	2QTS[7], 3S3W[2]
Closed	Narrow	Expanded	Yes	No	Yes	5WKU[8], 5WKV[8], 5WKX[5], 5WKY[5], 6AVE[8], 6VTL[6]
Closed	Narrow	Expanded	Yes	No	Yes	6X9H (ΔASIC1/JNJ-799760)

*Wide=D433 carboxyl oxygens>6 Å & G436 Cα> 8 Å (except 4NTY with 7.3 Å); Narrow=D433 carboxyl oxygens<4 Å & G436 Cα<4 Å (except 6VTK with 4.6 Å and 4.2 Å, respectively)

Supplementary Table 1. Comparison of key conformations of ΔASIC1/JNJ-799760 with those of published cASIC1 structures. The literature conventions are followed for orientations of the L414-N415 side chain swap and T84-R85 bond flip. For instance, these conformations in 5WKU are referred to as no swap and flipped, respectively.

References

1. Baconguis, I., Bohlen, C. J., Goehring, A., Julius, D. & Gouaux, E. X-ray structure of acid-sensing ion channel 1-snake toxin complex reveals open state of a Na^+-selective channel. *Cell* **156**, 717-29 (2014).
2. Dawson, R. J. et al. Structure of the Acid-sensing ion channel 1 in complex with the gating modifer Psalmotoxin 1. *Nat. Commun.* **3**, 936 (2012). https://doi.org/10.1038/ncomms1917
3. Baconguis, I. & Gouaux, E. Structural plasticity and dynamic selectivity of acid-sensing ion channel-spider toxin complexes. *Nature* **489**, 400-405 (2012).

4. Gonzales, E. B., Kawate, T. & Gouaux, E. Pore architecture and ion sites in acid-sensing ion channels and P2X receptors. *Nature* **460**, 599-604 (2009).
5. Yoder, N. & Gouaux, E. Divalent cation and chloride ion sites of chicken acid sensing ion channel 1a elucidated by x-ray crystallography. *PLoS One* **13**, e0202134, https://doi.org/ 10.1371/journal.pone.0202134 (2018).
6. Yoder, N. & Gouaux, E. The His-Gly motif of acid-sensing ion channels resides in a reentrant 'loop' implicated in gating and ion selectivity. *Elife*. **9**:e56527, https://doi:10.7554/eLife.56527 (2020).
7. Jasti, J., Furukawa, H., Gonzales, E. B. & Gouaux, E. Structure of acid-sensing ion channel 1 at 1.9 Å resolution and low pH. *Nature* **449**, 316-323 (2007).
8. Yoder, N., Yoshioka, C. & Gouaux E. Gating mechanisms of acid-sensing ion channels. *Nature* **555**, 397-401 (2018).
