# Supplementary material for: Molecular mechanism and structural basis of small-molecule modulation of the gating of acid-sensing ion channel 1
Source: Commun Biol. 2021 Feb 9;4:174. doi: 10.1038/s42003-021-01678-1 (PMC7873226; doi:10.1038/s42003-021-01678-1)
Supplement: Supplementary file 3 — Description of Additional Supplementary Files [file 42003_2021_1678_MOESM3_ESM.pdf]

Description of Additional Supplementary Files

File name: Supplementary Data 1

Description: The source data underlying the graphs and charts presented in the main figures.
